# Supplementary material for: Two-particle time-domain interferometry in the fractional quantum Hall effect regime
Source: Nat Commun. 2022 Oct 4;13:5863. doi: 10.1038/s41467-022-33603-3 (PMC9532452; doi:10.1038/s41467-022-33603-3)
Supplement: Supplementary file 1 — Supplementary Information [file 41467_2022_33603_MOESM1_ESM.pdf]

Supplementary Information for: "Two-particle time-domain interferometry in the Fractional Quantum Hall Effect regime"

Authors:

I. Taktak[1], M. Kapfer[1], J. Nath [1], P. Roulleau[1], M. Acciai[2], J. Splettstoesser[2], I. Farrer[3], D. A. Ritchie[4] and D.C. Glattli[1]*,

Affiliations:

[1] Université Paris-Saclay, CEA, CNRS, SPEC, 91191 Gif-sur-Yvette Cedex, France.

[2]Department of Microtechnology and Nanoscience - MC2, Chalmers University of Technology, S-412 96 Göteborg, Sweden.

[3]Department of Electronic and Electrical Engineering, University of Sheffield, Mappin Street, S1 3JD, UK

[4]Cavendish Laboratory, University of Cambridge, J.J. Thomson Avenue, Cambridge CB3 0HE, UK.

A. **Experimental Methods**

1. **Sample characteristics and fabrication**
2. **Measurement set-up**
3. **Visibility optimization**
4. **IQHE measurements at V_G=-0.27V**

B. **Electron-hole pair Interferometry**

1. **The Hanbury Brown Twiss phase**
2. **Channel mixing modeling**
3. **Comparison with MZI visibility loss due to similar channel mixing:**

C. **Supplementary references**

Supplementary Note A1. Sample characteristics and fabrication: samples are 2DEGs with electrons confined at the interface of high mobility epitaxially grown GaAs/GaAlAs heterojunctions at 90 nm below the surface. The low temperature zero field mobility is $2.10^6 cm^2 s^-$

$^{1}V^{-1}$ and the electron density is n_s=1,11 $10^{15}m^{-2}$. For this density, the bulk filling factor ν_B=2/5 corresponds to a magnetic field of ≈11.2 Tesla. Ohmic contacts are realized by evaporating 125 nm Au, 60 nm Ge, 4 nm Ni followed by annealing at 470°C. A shallow mesa etching (H3PO4 phosphoric acid, time 4 minutes) defines the sample. The QPC gates are realized by e-beam lithography, see Fig.S1 for a SEM image of the sample used. The edge channel length between each contacts and the center of the QPC is about 18μm. .

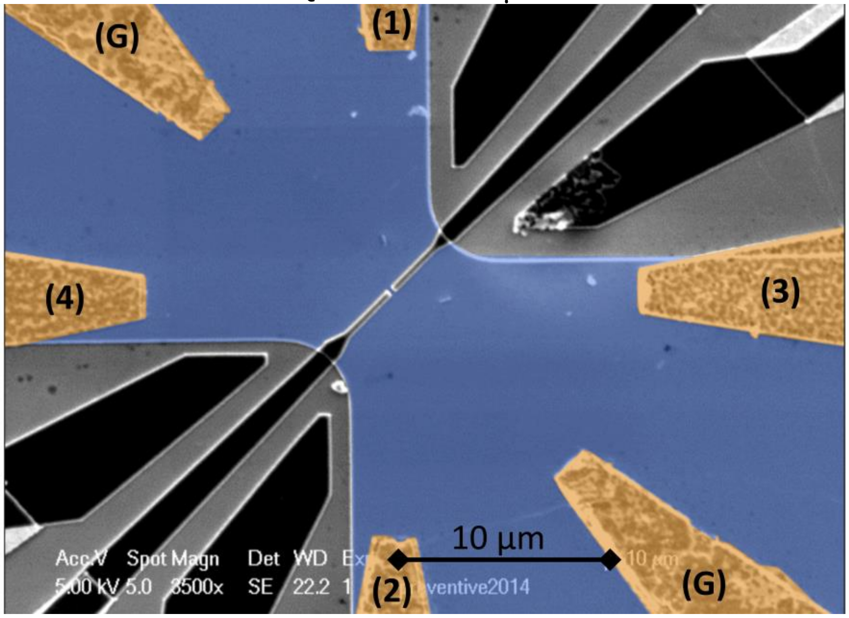

Figure S1: SEM view of the sample used. Yellow areas are ohmic contacts, blue aeras denote the unetched part of 2DEG mesa. A 10um black bar indicates the scale.

Supplementary Note A2. Measurement set-up: an ultra-low temperature cryo-free dilution refrigerator with a 20 mK base temperature from CryoConcept is used as in [1,2]. It is equipped with a dry superconducting coil able to reach 14.5 Tesla. Ultra-low-loss dc-40GHz microwave cables, same as in ref. [1,2], bring the room temperature microwave excitation from an Agilent N5183A RF source to a Printed Circuit Board (PCB). The RF power of the microwave source, given in the main text is attenuated by fixed 63dB cold attenuators and extra losses in the cryogenic coaxial cables. Coplanar waveguides designed by CST microwave Studio[R] etched on the PCB bring the two radiofrequency excitations to ohmic contact (1) and (2) of the sample, see Fig.1(f) and Fig.S1. Noise measurements are obtained by separately converting the transmitted and reflected current fluctuations into voltage fluctuations at contact (3) and (4) respectively in parallel to a R-L-C resonant circuit tuned to 2,2 MHz frequency and bandwidth ≈ 150 kHz, with R=20kOhms. Note that an effective resonant circuit resistance $R_{eff.} = RR_L/(R + R_L) \approx$ 6.5kOhms is found instead of 20kOhms due to inductance loss, giving a shunt resistance $R_L = (L2\pi f_0)^2/r$ in parallel to R, where r=15 Ohms is the series resistance of the inductance. Finally, the Q factor of the RLC resonant circuit is given by the ratio of the parallel resistance $R_{eff}//R_{Hall}$ to the characteristic impedance $\sqrt{\frac{L}{C}}$, where R_{Hall} is the Hall resistance of the sample. The voltage fluctuations are amplified by two home-made cryogenic amplifiers with 0.22 nV/$Hz^{1/2}$ input noise at low temperature, followed by low noise room temperature amplifiers. The amplified fluctuations are passed through Chebyshev filters and then sent to a fast 20Ms/s digital acquisition card

(ADLink 9826) while a PC provides real-time computation of the cross-correlation spectrum. Absolute Noise calibration is done by recording the equilibrium Johnson Nyquist noise when varying the temperature from 20mK to 200mK. Differential Conductance measurements giving the transmission and reflection are made by applying a low frequency AC voltage, frequency 270Hz, and μV amplitude voltage to contact (1) and sending the amplified AC voltage from contacts (3) and (4) to two Lock-in amplifiers. The low frequency measurement accuracy is mostly limited by the large 1/f noise of the cryogenic HEMT (white noise cross-over at ≈1MHz). The shot noise accuracy is limited by the input white noise of the amplifier and time averaging. For ν_B=2/5, the 20kOhm resistor and the ≈5kOhm inductance the effective RLC parallel resistance R_{eff}=6.5kOhms in parallel with the bulk Hall resistance converts the input noise of 220pV/Hz$^{1/2}$ into 1.410^{-27}A^2/Hz equivalent current noise power. Using cross-correlation and noise averaging during the measurement time τ_m =3s with ≈150kHz effective detection bandwidth, the accuracy of a raw noise data point is +/- 2 10^{-30}A^2/Hz.

Supplementary Note A3. Visibility optimization:

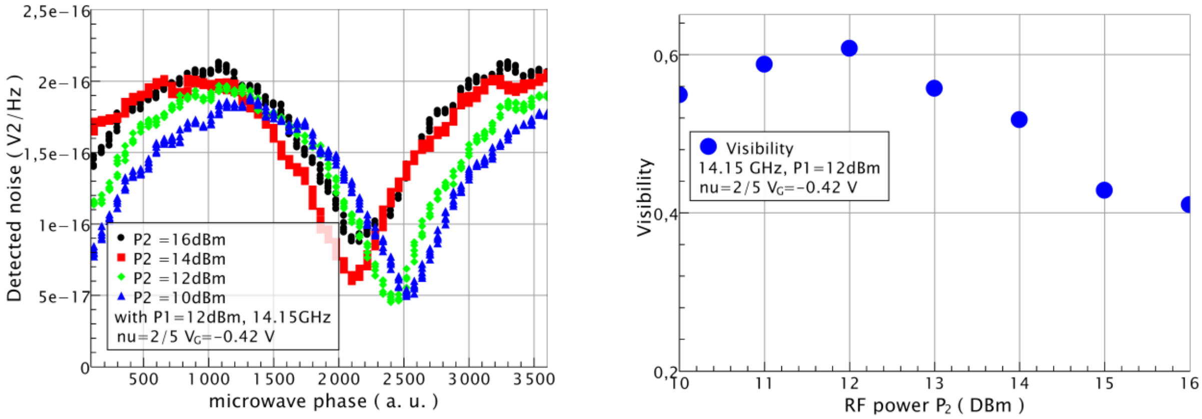

Fig. S2: Visibility optimization.

The left graph shows noise measurements versus time-delay for various RF source powers P_2 sent to contact (2) while the power sent to contact (1) is P_1= 12dBm. The equality of the actual V_{ac} amplitudes transmitted on contacts (1) and (2) is obtained for the maximum of the visibility. Right graph: visibility versus power P_2 in dBm.

In the main text, all noise interference formulas are given for equal RF amplitudes applied on the injecting contacts. As the microwave transmission of the RF-lines is not accurately known at low temperature and may suffer from unwanted microwave reflection in connectors linking different temperature stages or suffer from uncontrolled dissipation, it is best to find a way to determine in situ the equality of the RF amplitudes. This is done by fixing the microwave amplitude on contact (1) and varying the amplitude on contact (2) while measuring the noise versus time-delay. The maximum of visibility signals equal RF amplitudes and allows to fix P2, here about -0.5dB lower than P1 to ensure perfect equality of the amplitudes on the sample.

Supplementary Note A4. IQHE measurements at V_G=-0.27V

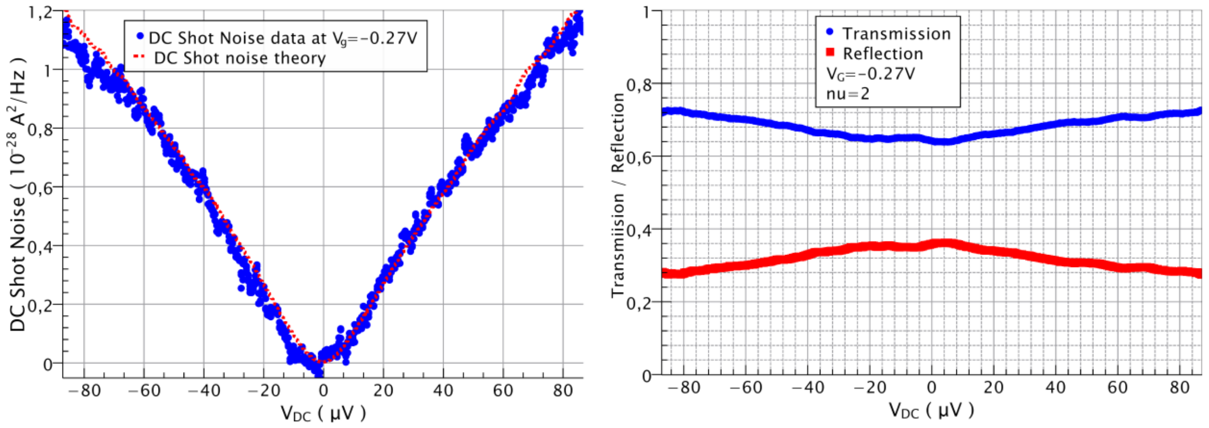

Fig. S3: right: transmission and reflection probability for the partial transmission of the inner edge channel versus bias voltage at V_G=-0.27V and filling factor 2. Left: DC shot noise measured in the same conditions. The red dashed curve is calculated using the DC shot noise theoretical expression (4) in which a weak energy dependent transmission deduced from the right graph is included.

Here we present measurements which are complementary to the ones presented in the main text. The mean inner edge channel reflection is R=0.3 for V_G=-0.27V. Figure S3 shows a slight non-linearity of the transmission versus DC voltage bias. The DC shot noise trace (blue filled circles) is well described by the standard DC shot noise formula, equation (4), using the measured transmission.

Figure S4 shows the two-particle interference shot noise for 5dBm and 8dBm RF source power at frequency 14.15GHz (blue filled circles). The red dashed line is a fit using the heuristic equation (3) for a constant transmission D=0.7 and AC voltage amplitude V_{ac}=33.5 and 41 μV for respectively 5 and 8 dBm. The extracted values are close to the ones deduced with similar RF power at the other gate voltage -0.20V. The black dashed line is a fit using a model of channel mixing, see equation (S6) below.

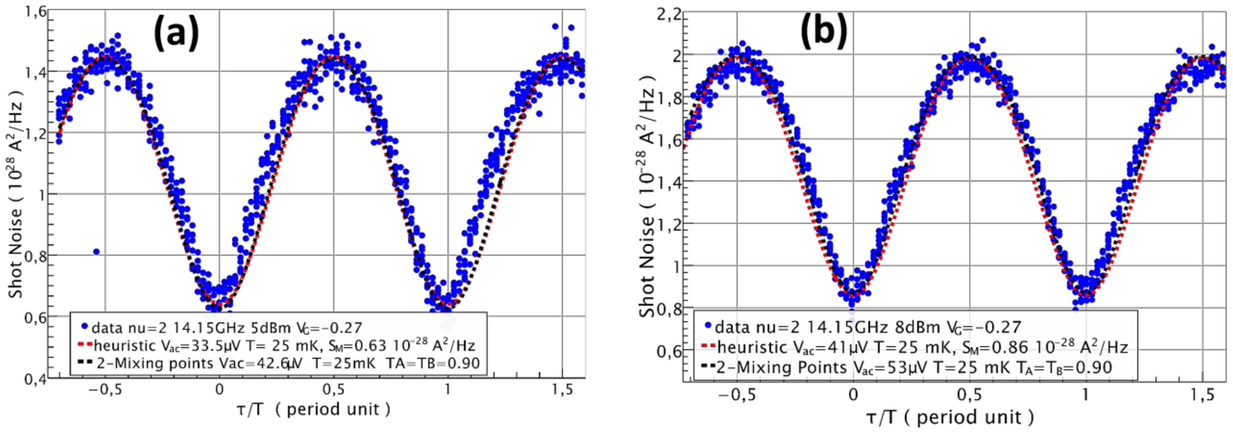

Fig. S4: Shot noise versus time-delay measured for 14.15GHz microwave excitation and RF source powers 5dBm (a) and 8 dBm (b) respectively at V_G=-0.27V (blue filled circles). The comparison with the heuristic equation (3) including a noise offset S_M=0.63 and 0.86 10^{-28} A^2/Hz is shown as a red dashed curve. Comparison with equation (S6) for a model of two symmetric mixing points (strength $R_A=R_B=0.10$, $\Delta\tau=\tau_i-\tau_o$=44ps) placed on both input channels is shown as a black dashed curve. A constant QPC transmission 0.7 and a 25mK temperature are used to fit the data.

Supplementary Note B1. The Hanbury Brown Twiss phase:

We consider zero temperature for simplicity and no channel mixing such that the scattering of a single channel by the QPC only occurs. Electrons emitted by the reservoir at energy ε and experiencing the AC voltage in contacts (1) and (2) are put in a quantum superposition of states with energies $\varepsilon_{+/-} = \varepsilon \pm hf$. For low AC amplitude, the amplitude of probability to absorb/emit a single photon is $p_{\pm 1} = \pm\left(\frac{eV_{ac}}{2hf}\right)^2$ for contact (1) and $p_{\pm 1}e^{i\Delta\phi} = \pm\left(\frac{eV_{ac}}{2hf}\right)^2 e^{i\Delta\phi}$ for contact (2) where $\Delta\phi=2\pi f\tau$ is the voltage phase shift. Higher photon number processes are discarded for $\left(\frac{eV_{ac}}{2hf}\right)^2 \ll 1$. The many-particle state incident on the scattering region is then [3]:

$$|in\rangle = |F\rangle + \int_0^{hf} d\varepsilon\, p_1 \left(\hat{a}_1^\dagger(\varepsilon)\hat{a}_1(\varepsilon_-) + e^{i\Delta\phi}\hat{a}_2^\dagger(\varepsilon)\hat{a}_2(\varepsilon_-)\right)|F\rangle \tag{S1}$$

where $|F\rangle = \prod_{\varepsilon<E_{F,\alpha=1-4}} \hat{a}_\alpha^\dagger(\varepsilon)|0\rangle_\alpha$ is the ground state formed by the filled Fermi sea of all leads and $\hat{a}_\alpha^\dagger(\varepsilon)$ the fermionic creation operator acting on the occupation states of contacts α=1,2.

Denoting $\widetilde{\hat{a}_\alpha}(\varepsilon) = \hat{a}_\alpha(\varepsilon) + p_1\hat{a}_\alpha(\varepsilon - hf) + p_{-1}\hat{a}_\alpha(\varepsilon + hf)$ the annihilation operator describing, to lowest order in single photon absorption/emission probability, the electrons emitted by lead (α) and scattered in energy by the AC potential, the operators describing the states of electrons entering in contacts (3) and (4) are:

$$\hat{b}_3(\varepsilon) = s_{31}\widetilde{\hat{a}_1}(\varepsilon) + s_{32}\widetilde{\hat{a}_2}(\varepsilon) \tag{S2a}$$

$$\hat{b}_4(\varepsilon) = s_{41}\widetilde{\hat{a}_1}(\varepsilon) + s_{42}\widetilde{\hat{a}_2}(\varepsilon) \tag{S2b}$$

The many-particle outgoing state is then:

$$|out\rangle = |0\rangle + \int_0^{hf} d\varepsilon\, p_1 \Big[s_{31}s_{31}^*\hat{b}_3^\dagger(\varepsilon)\hat{b}_3(\varepsilon_-) + s_{41}s_{41}^*\hat{b}_4^\dagger(\varepsilon)\hat{b}_4(\varepsilon_-) + s_{31}s_{41}^*\hat{b}_3^\dagger(\varepsilon)\hat{b}_4(\varepsilon_-) + s_{41}s_{31}^*\hat{b}_4^\dagger(\varepsilon)\hat{b}_3(\varepsilon_-) + e^{i\Delta\phi}\Big(s_{32}s_{32}^*\hat{b}_3^\dagger(\varepsilon)\hat{b}_3(\varepsilon_-) + s_{42}s_{42}^*\hat{b}_4^\dagger(\varepsilon)\hat{b}_4(\varepsilon_-) + s_{32}s_{42}^*\hat{b}_3^\dagger(\varepsilon)\hat{b}_4(\varepsilon_-) + s_{42}s_{32}^*\hat{b}_4^\dagger(\varepsilon)\hat{b}_3(\varepsilon_-)\Big)\Big]|0\rangle \tag{S3}$$

The terms of the form $\hat{b}_\alpha^\dagger(\varepsilon)\hat{b}_\alpha(\varepsilon_-)$ describe neutral excitations in the output leads and do not contribute to current noise at zero frequency. The only terms contributing to the zero frequency cross-correlated fluctuations of leads (3) and (4) are of the form $\hat{b}_\alpha^\dagger(\varepsilon)\hat{b}_{\beta\neq\alpha}(\varepsilon_-)$. Namely:

$$p_1\left[s_{31}s_{41}^* + e^{i\Delta\Phi}s_{32}s_{42}^*\right]\hat{b}_3^\dagger(\varepsilon)\hat{b}_4(\varepsilon_-) + p_1\left[s_{41}s_{31}^* + e^{i\Delta\Phi}s_{42}s_{32}^*\right]\hat{b}_4^\dagger(\varepsilon)\hat{b}_3(\varepsilon_-) \tag{S4}$$

The first term corresponds to the interference of electron-hole pairs separately created in lead (1) and (2) and scattered as an electron in lead (3) and a hole in lead (4), as shown in figure 1(b) and (c) of the main text. The second term describes the interference leading to an electron created in lead (4) and a hole in lead (3). These terms leads to equation (1) of the main text.

Defining $s_{31} = te^{i\varphi_{31}}$, $s_{42} = te^{i\varphi_{42}}$, $s_{41} = (ir)e^{i\varphi_{41}}$, $s_{32} = (ir)e^{i\varphi_{32}}$, with $\varphi_{\beta\alpha}$ denoting the electronic phase accumulated while propagating from contact (α) to contact (β) and using the local transmission and reflection amplitudes t and (ir) for the QPC, we see that the interference allows us to probe the so-called HBT phase [3] $\chi = arg(s_{13}^* s_{32} s_{24}^* s_{41}) = \pi + (\varphi_{32} + \varphi_{41}) - (\varphi_{31} + \varphi_{42})$ using the controlled time-delay of the sinewave sources. Expression (S4) vanishes for $\Delta\Phi = \chi - \pi$. The phase χ, not accessible to DC transport and DC noise measurement, is the relevant phase of the two-particle dynamical interferometer and plays a role similar to the phase of single particle interferometers. Note that the actual value is not important because it is sample dependent like the different arms of an MZI. Even in the original work of Ref. [3], the actual value of the phase χ was not presented as fundamental.

Let us discuss in more details the interference process. One can interpret the first term in brackets in equation (S4) in the following way: when an electron-hole pair is created in lead (1) the 2-particle probability amplitude to have an electron in lead (3) and hole in lead (4) is $p_1 s_{31} s_{41}^*$. Similarly, when an electron-hole pair is created in lead (2) the 2-particle probability amplitude to have an electron transmitted in lead (3) and hole backscattered in lead (4) is $p_1 e^{i\Delta\Phi} s_{32} s_{42}^*$. As these two processes are not distinguishable, one has to add the probability amplitude of each process, hence the term in brackets. The second term in brackets in (S4) represents a similar two-particle interference but with a hole in lead (3) and an electron in lead (4). The +/-e charge fluctuations generated in output leads by these two processes have equal probability. This leads to the noise power density given by equation (1).

One can extend the two-particle interference processes to the FQHE regime. When an electron-hole pair is created, the transmitted electron (hole) is transmitted as a whole, charge e (-e) while, in contrast to the IQHE regime, the standard backscattering is replaced by a partial backscattering of the incoming charge. One finds a split hole (electron) transmitted as a charge (m-1)/me (-(m-1)/me) and backscattered as a charge e/m (-e/m). This leads to equation (7) using the weak backscattering of the scattering amplitudes. Equation (7) quantitatively agrees, in this limit, with a more involved interacting electron theory.

Supplementary Note B2. Channel mixing modeling:

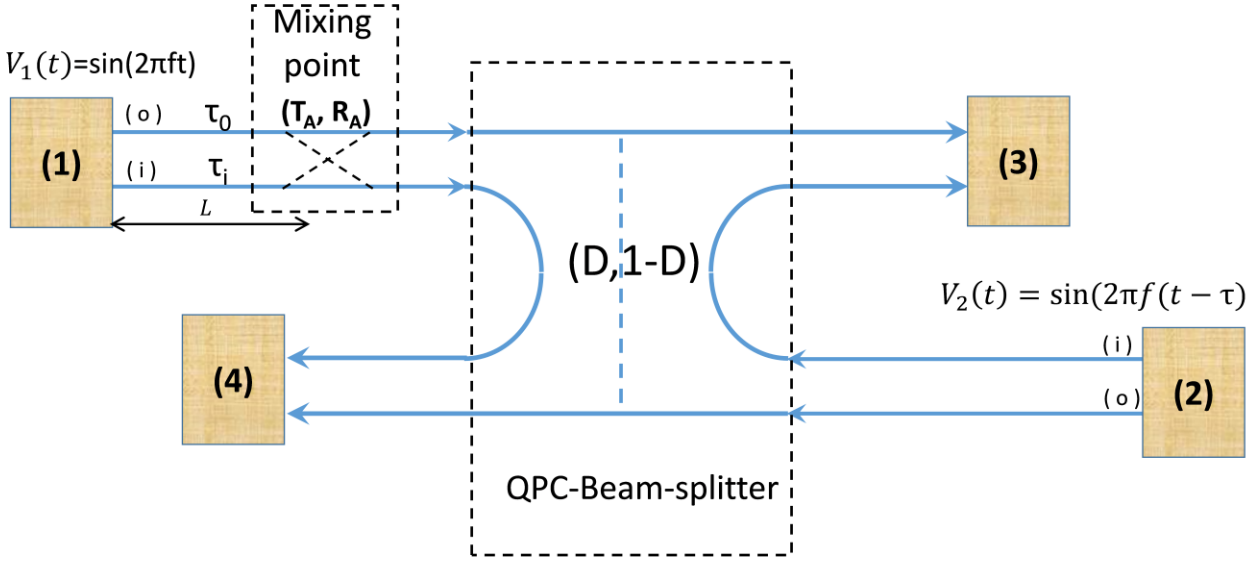

Figure S5: elastic channel mixing. The QPC beam splitter is aimed at partitioning the outer edge channel only, transmission D. However, a localized scatterer situated at distance L from the injecting contact (1) allows tunneling between the outer and inner edges. The probability of inter-edge tunneling is R_A. T_A=1-R_A is the probability of no mixing. For different outer and inner edge channel propagation velocities V_0 and V_i , electrons on the outer and inner edge have different dynamical phase $\varphi_o=2\pi f\tau_o$ and $\varphi_i=2\pi f\tau_i$. This generates photo-assisted shot noise making electrons arriving at the upper left of the QPC beam splitter noisy, reducing the visibility of the two-particle interference. In other words, the mixing point allows the inner edge channel to know "which path" the interfering electron-hole pairs are coming from, spoiling quantum coherence. Similar physics holds when the QPC partitions the inner edge while transmitting the outer edge.

Figure S5 schematically shows how one can model the effect of channel mixing. We consider only a single mixing point along one input edge channel (the left input). Generalization to more mixing points both on the left and right side of the central QPC beam-splitter is straightforward and gives similar qualitative effects, see Ref.[4]. Being interested in bulk filling factors ν=2 and 2/5, we consider only co-propagating edge channels.

In the absence of mixing, the inner edge channel is spectator and the noise of lead (3) or (4) measures the two-particle interference of indistinguishable electron-hole pairs photo-created in the outer at input leads (1) and (2).The visibility is expected to be 100%. However a finite mixing allows the inner edge to acquire information on which path an electron-hole pair comes from and the visibility is reduced. Note that this requires that the wave-function of particles propagating on channel (o) and (i) can be distinguished. This is indeed so if the propagation time $\tau_{i,o}$ in the inner and outer edge are different such that the dynamical phases $\varphi_{i/o}$ are different. A full calculation using standard Floquet scattering theory can be found in [4]. For a single scattering point, as considered in figure S5, one finds the shot noise S_I:

$$-S_{I_3 I_4}(\tau) = 2\frac{e^2}{h}hfD^2(T_A R_A)\left(\frac{eV_{ac}2\sin(2\pi f(\Delta\tau))}{hf}\right)^2 + T_A 2(e^*)^2 fD(1-D)\left(\frac{e^*V_{ac}}{2hf}\right)^2(1-\cos(2\pi f(\tau-\tau_i)) + R_A 2(e^*)^2 fD(1-D)\left(\frac{e^*V_{ac}}{2hf}\right)^2(1-cos(2\pi f(\tau-\tau_0))) \quad \text{(S5)}$$

Where $\Delta\tau=\tau_o-\tau_i$ and we have assumed for simplicity that the propagation time from the scatter to the QPC is negligible. Including finite propagation time towards the QPC can be taken into account by a proper redefinition of the time delay τ.

The effect of mixing is twofold: -1) the generation of an extra noise given by the first term of equation (S5) ; -2) the smearing of the interference as τ cannot make the last two terms simultaneously vanish unless $\tau_o=\tau_i=\tau$.

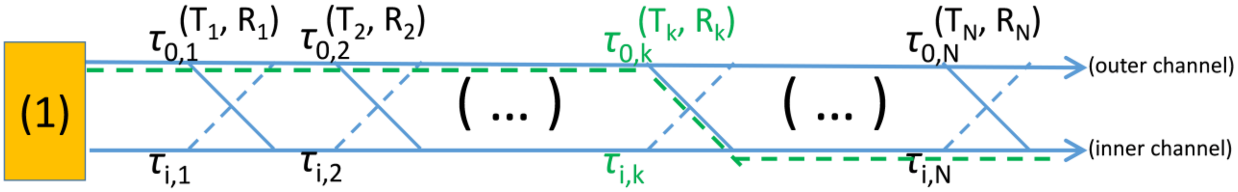

Figure S6: multiple inter edge channel tunneling points. $\tau_{o,k}$ and $\tau_{i,k}$ denote the propagation time between point (k) and (k-1). For weak inter-edge tunneling, $R_{k=1,N} \ll 1$, and, now, the inner channel partitioned by the QPC, one can add in the HBT interference noise the independent contribution of each tunneling point (k) weighted by the probability R_k and using the time delay $\tau_k = \sum_{q\le k}\tau_{o,q} + \sum_{q>k}\tau_{i,q}$ corresponding to the propagation along the dashed green path describing the tunneling from the outer edge to the inner edge at point (k). These contributions lead to the smearing of the HBT interference and loss of visibility.

The model can be extended to two mixing points of strength R_A and R_B placed on both input channels at a distance L from the injecting contacts, see [4]. Here we give the expression for symmetrically placed mixing points of equal strength. This expression is used to fit the data of figure S4 (black dashed curves).

$$-S_{I_3I_4} = 2\frac{e^2}{h}hf\left[D(1-D)\left((T_A^2+R_A^2)S(\tau)+T_AR_A(S(\tau+\Delta\tau)+S(\tau-\Delta\tau)\right)+2D^2T_AR_AS(\Delta\tau)\right] \quad \text{(S6)}$$

Here, τ is the controlled time delay between the AC sources, $\Delta\tau = |\tau_i - \tau_o| = \left|\frac{L}{V_i} - \frac{L}{V_o}\right|$ is the propagation time difference for particles travelling from the contact to the mixing point with velocity V_i and V_o for respectively the inner and outer edge, T_A=1-R_A and $S(\tau) = \sum_l\ l J_l^2(\frac{e^*V_{ac}}{hf} 2\sin\left(\frac{\Delta\Phi'}{2}\right)\left(\coth\left(\frac{lhf}{2k_BT}\right)-\frac{2k_BT}{lhf}\right)$. The last term in (S6), independent of τ, originates from the noise generated at each mixing point and plays the role of S_M in the heuristic formula, the first three terms are interference terms. The shift by +/-Δτ with respect to τ in the argument of $S(\tau)$ contributes to the smearing of the HBT interference. Figure S4 uses equation (S6) to fit the experimental data. A moderate 10% mixing strength (R_A=0.1) is found. This is consistent with a channel mixing length observed in the literature [5-7]. For L necessarily smaller than the 18μm input channel length, a propagation time difference of 44ps suggests an outer edge channel velocity >2.4 10^5m/s dominating the inner edge velocity. This is consistent with the outer and inner edge channel velocities reported for filling factor 2. A fit of the data of similar quality is obtained using two different mixing parameters T_A=0.92 and T_B=0.86 while keeping same V_{ac} and Δτ values.

The smearing of the interference pattern is probably the most serious loss of visibility if multiple tunneling points occur. For example, let us consider multiple mixing points in one arm, say the left, as suggested in Fig S6, where we choose to partition the inner edge. To first order of inter-edge tunneling probabilities $\{R_p\}$, we are left with a sum of terms corresponding to tunneling paths (k) where an electron emitted from (o) has been transferred to the input edge (i) at the kth tunneling point corresponding to the propagation time $\tau_k = \sum_{q\le k}\tau_{o,q} + \sum_{q>k}\tau_{i,q}$ and contributing by $R_k 2(e^*)^2 fD(1-D)\left(\frac{e^*V_{ac}}{2hf}\right)^2(1-cos(2\pi f(\tau-\tau_k)))$ to the two-particle noise.

For stronger mixing $R_{k=1,N}$, second order inter-tunneling probabilities (like the case where an electron emitted in channel (i) visit once channel (o) and returns back to channel (i) to contribute to the interference) add more paths with different propagation times. This further smears the original interference pattern. In a more realistic description one has to consider multiple mixing points in both left and right input leads. This contributes to more loss of visibility.

The following graph, Fig S7, compares the visibility obtained for a single mixing point approach and that obtained by considering the continuous limit of an ensemble average of weak mixing points (multiple tunneling between outer and inner edge channel are neglected). We observe that the two curves are very similar, but in order to compare the continuous mixing point (red dashed) curve with the single mixing point (blue) curve, a slight increase (13%) of the mixing strength R_A has to be done. One can conclude that, for weak mixing, a single mixing point and a continuous distribution of mixing points give equally a good qualitative and quantitative representation of the data.

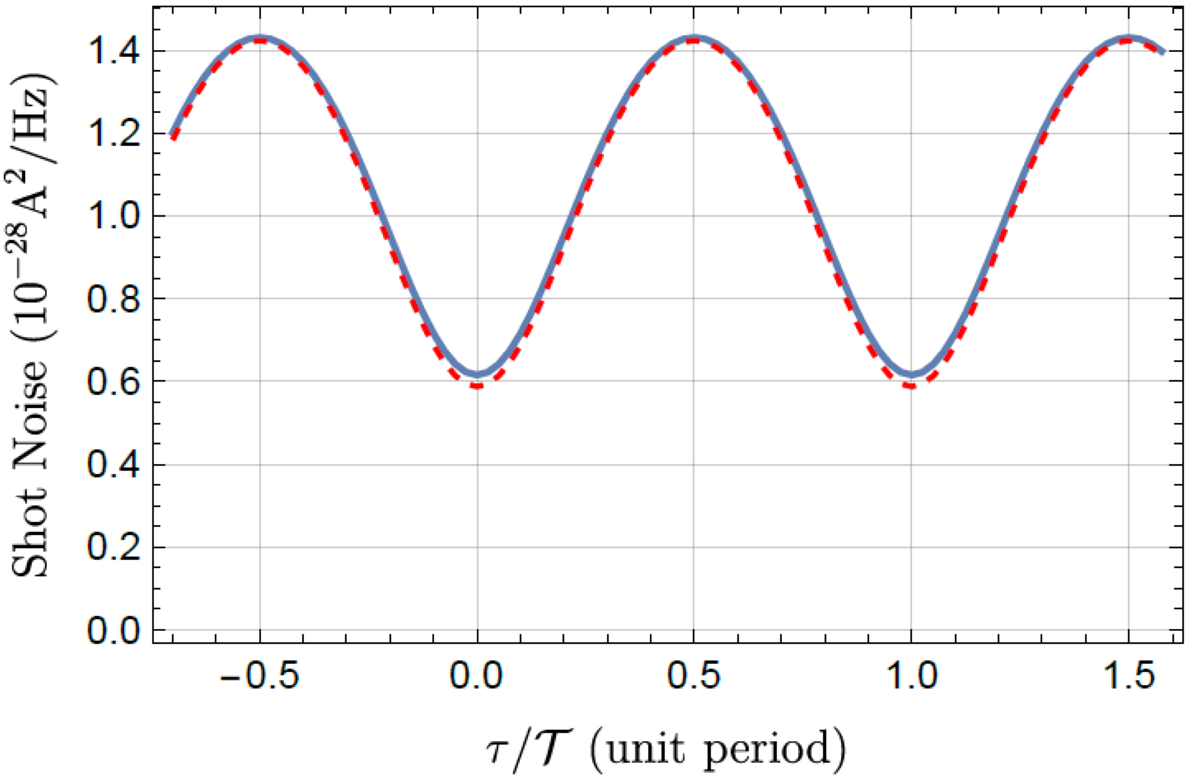

Figure S7: Blue curve: fit of the experimental data of Fig. S4(a) using single mixing points of strength R_A=0.1, eV_{ac}/hf = 0.727, Δτ=44ps and 25mK temperature. The red dashed line curve is the calculation of an ensemble average of mixing points but the strength has been increased to R_A=0.13 while other parameters are kept constant.

Supplementary Note B3. Comparison with MZI visibility loss due to similar channel mixing:

A direct comparison of the loss of coherence in a MZI and that observed in our experiment is not straightforward. While in a MZI, channel mixing alone is enough to induce a loss of visibility, in the present experiment having both channel mixing and different channel velocity propagation are required to explain a reduction of visibility. Nevertheless, in order to provide a tentative comparison, we present here a calculation of the MZI loss of visibility expected for a mixing strength equal to the one observed in our two-particle dynamical interference measurements.

Here we consider the effect of channel mixing on the visibility of a Mach-Zehnder interferometer. Then we will compare, for the same mixing channel, the loss of visibility for two-particle HBT interference and MZI interference.

We consider a MZI at filling factor ν=2. Interference is performed on the inner channel using two quantum point contacts of transmission T_1 and T_2. An outer edge channel is copropagating along the inner edge. In absence of inner/outer edge channel mixing, the output current I_i found in the inner edge is:

$$I_i = \frac{e^2}{h} V_{DC}\left(T_1 T_2 + R_1 R_2 - 2\sqrt{T_1 T_2 R_1 R_2}\cos(\phi)\right) \quad \text{(S7)}$$

Where ϕ is the phase including a possible arm length difference and an Aharonov-Bohm flux, and the dependence in energy has been neglected for simplicity. Let us now consider a single mixing point, located at some position (A) on the upper arm and denote the tunneling probability between inner and outer edge channel as R_A=1-T_A. By analogy with the case of our dynamical HBT interference, we will consider that both inner and outer edges are fed by the same ohmic contact at potential V_{DC} while all other contacts are at zero potential.

Let us now calculate the current I_i in the inner output edge. It is made of two contributions. A first contribution to I_i is an interference contribution $I_{i(i)}$ due to electrons injected in the inner edge by the polarized contact:

$$I_{i(i)} = \frac{e^2}{h} V_{DC}\left((T_1 T_A) T_2 + R_1 R_2 - 2\sqrt{(T_1 T_A) T_2 R_1 R_2}\cos(\phi)\right) \quad \text{(S8)}$$

A second contribution to I_i is a non-interference contribution $I_{i(o)}$ due to electrons emitted in the outer edge by the polarized contact:

$$I_{i(o)} = \frac{e^2}{h} V_{DC}(R_A T_2) \quad \text{(S9)}$$

The total inner edge output current is:

$$I_i = \frac{e^2}{h} V_{DC}\left(R_A T_2 + (T_1 T_A) T_2 + R_1 R_2 - 2\sqrt{(T_1 T_A) T_2 R_1 R_2}\cos(\phi)\right) \quad \text{(S10)}$$

The interference visibility for a single mixing point $V_{i,1}$ is:

$$V_{i,1} = \frac{2\sqrt{(T_1 T_A) T_2 R_1 R_2}}{R_A T_2 + (T_1 T_A) T_2 + R_1 R_2} \quad \text{(S11)}$$

In the case of two impurities, one of strength R_A in the upper arm and the other one of strength R_B in the lower arm, we have similarly an interfering term:

$$I_{i(i)} = \frac{e^2}{h} V_{DC}\left((T_1 T_A)T_2 + (R_1 T_B)R_2 - 2\sqrt{(T_1 T_A)T_2 (R_1 T_B) R_2}\cos(\phi)\right) \qquad \text{(S12)}$$

And a non-interfering term similar to the previous case:

$$I_{i(o)} = \frac{e^2}{h} V_{DC}(R_A T_2) \qquad \text{(S13)}$$

The interference visibility for two mixing points $V_{i,2}$ is:

$$V_{i,2} = \frac{2\sqrt{(T_1 T_A)T_2 (R_1 T_B) R_2}}{R_A T_2 + (T_1 T_A)T_2 + (R_1 T_B) R_2} \qquad \text{(S14)}$$

For the mixing strength R_A=R_B = 0.1 deduced from figure S4 the two-particle dynamical HBT interference gives a 40% visibility. For the single-particle Mach-Zehnder interference, according to the above calculation, equation (S14), a visibility of 90% is expected for the best tuning of the MZI (T_1=T_2=0.5).

D. Supplementary references

[1] J. Dubois, T. Jullien, F. Portier, P. Roche, A. Cavanna, Y. Jin, W. Wegscheider, P. Roulleau and D. C. Glattli, “Minimal-excitation states for electron quantum optics using levitons”, *Nature* **502**, 659–663 (2013)

[2] M. Kapfer, P. Roulleau, M. Santin, I. Farrer, A. Ritchie and D. C. Glattli "A Josephson relation for fractionally charged anyons", Science, Vol 363, pp. 846-849 (2019)

[3] V. S. Rychkov, M. L. Polianski, and M. Büttiker, “Photon-assisted electron-hole shot noise in multiterminal conductors”, Phys. Rev. B 72, 155326 (2005)

[4] M. Acciai, P. Roulleau, I. Taktak, D. C. Glattli, and J. Splettstoesser, “Influence of channel mixing in fermionic Hong-Ou-Mandel experiments”, Phys. Rev. B 105, 125415 (2022).

[5] Y. Takagaki, K. J. Friedland, J. Herfort, H. Kostial, and K. Ploog, “Inter-edge-state scattering in the spinpolarized quantum Hall regime with current injection into inner states," Phys. Rev. B 50, 4456-4462 (1994)

[6] Y. Acremann, T. Heinzel, K. Ensslin, E. Gini, H. Melchior, and M. Holland, “Individual scatterers as microscopic origin of equilibration between spin-polarized edge channels in the quantum Hall regime," Phys. Rev. B 59, 2116-2119 (1999)

[7] G. Muller, D. Weiss, S. Koch, K. von Klitzing, H. Nickel, W. Schlapp, and R. Losch, “Edge channels and the role of contacts in the quantum Hall regime," Phys. Rev. B 42, 7633 (1990)
